# MRI correlates of motoneuron loss in SMA

**DOI:** 10.1007/s00415-022-11326-1

**Published:** 2022-10-01

**Authors:** Alina Sprenger-Svačina, Johannes Haensch, Kilian Weiss, Nils Große Hokamp, David Maintz, Marc Schlamann, Gereon R. Fink, Natalie Schloss, Kai Laukamp, Gilbert Wunderlich, Helmar C. Lehmann, Thorsten Lichtenstein

**Affiliations:** 1 Department of Neurology, Medical Faculty and University Hospital of Cologne, University of Cologne, Kerpener Straße 62, 50937 Cologne, Germany; 2 Institute for Diagnostic and Interventional Radiology, Faculty of Medicine and University Hospital Cologne, University of Cologne, Cologne, Germany; 3 Philips GmbH Market DACH, Hamburg, Germany; 4 Institute of Neuroscience and Medicine (INM-3), Research Centre Juelich, Juelich, Germany

**Keywords:** Spinal muscular atrophy, Proton-density fat fraction, Water *T*_2_ mapping, DTI, Neuromuscular disease

## Abstract

**Background:**

Magnetic resonance imaging (MRI) is currently explored as supplemental tool to monitor disease progression and treatment response in various neuromuscular disorders. We here assessed the utility of a multi-parametric magnetic resonance imaging (MRI) protocol including quantitative water *T*_2_ mapping, Dixon-based proton density fat fraction (PDFF) estimation and diffusion tensor imaging (DTI) to detect loss of spinal motor neurons and subsequent muscle damage in adult SMA patients.

**Methods:**

Sixteen SMA patients and 13 age-matched controls were enrolled in this prospective, longitudinal study. All participants underwent MRI imaging including measurements of Dixon-based PDFF and DTI of the sciatic nerve. SMA patients furthermore underwent measurements of muscle water *T*_2_ (*T*_2w_) of the biceps femoris muscle (BFM) and quadriceps femoris muscle (QFM). Ten participants returned for a second scan six months later. MRI parameter were correlated with clinical data. All patients were on nusinersen treatment.

**Results:**

There were significantly higher intramuscular fat fractions in the BFM and QFM of SMA patients compared to healthy controls at baseline and after 6 months. Furthermore, T2 values significantly correlated positively with intramuscular fat fractions. The Hammersmith functional motor scale significantly correlated with the QFM’s intramuscular fat fractions. DTI scans of the sciatic nerve were not significantly different between the two groups.

**Conclusion:**

This study demonstrates that, water *T*_2_ mapping and Dixon-based PDFF estimation may distinguish between adult SMA patients and controls, due to massive intramuscular fat accumulation in SMA. More extensive long-term studies are warranted to further evaluate these two modalities as surrogate markers in SMA patients during treatment.

## Introduction

Spinal muscular atrophy (SMA) is characterized by degeneration of the spinal cord’s alpha motor neurons, resulting in severely disabling progressive muscle weakness. SMA is an autosomal-recessive disorder caused by mutations in the survival motor neuron 1 gene, *SMN1*, most commonly due to deletion or mutation localized at 5q11.2-q13.3. Four phenotypes (SMA1–4) are characterized depending on the age of onset and motor function achieved: very weak infants unable to sit unsupported (SMA1), non-ambulant patients able to sit independently (SMA2) up to ambulant patients with childhood (SMA3), and adult-onset SMA (SMA4). [1]. For adult SMA patients, two splicing modifiers, Nusinersen and Risdiplam are currently approved for treatment [2–5].

Disease progression in adult SMA is usually monitored by clinical tests, i.e., Hammersmith Functional Motor Scale (HFMS) and its expanded version (HFSME), the Revised Upper Limb Module (RULM), or the 6-min walk test (6MWT) [6]. However, these clinical tests have limitations. For example, the HFMS(E) is time-consuming, biased by fatigue, and non-linear [7]. Therefore, non-invasive biomarkers could be useful to monitor nerve and muscle changes and to measure the treatment response in SMA.

Many studies explored the utility of magnetic resonance imaging (MRI) as diagnostic and surrogate markers for motor neuron diseases including SMA. Patients with SMA show fatty replacement and muscle atrophy in T1-weighted images [8]. By the use of the so-called Dixon-based proton density fat fraction (PDFF) estimation, Otto and colleagues demonstrated in a small cross-sectional study that SMA patients have sixfold higher fat fractions in the thigh muscles than healthy controls [9]. Likewise, two other studies assessed muscle fat infiltration of thigh muscles in smaller cohorts of non-treated SMA patients [10, 11]. Bonati et al. [11] carved out a significant, almost tenfold higher muscle fat content in SMA3 patients than healthy controls with high reproducibility after 6 months. Additionally, water *T*_2_ (*T*_2w_) mapping has been used as a biomarker in different neuromuscular diseases, as it reflects pathophysiological changes of skeletal muscle tissue and is sensitive to different processes, such as inflammation, edema, and myocytic lesions [9, 12, 13].

Muscle MRI can be supplemented by nerve diffusion tensor imaging (DTI), revealing information about nerve damage and regeneration [14–18]. Previous studies in other neuromuscular and neuropathy disorders found a decreased fractional anisotropy (FA) in the sciatic nerve compared to controls [14, 19]. So far, studies in SMA patients only investigated the FA in muscle tissue showing increased values compared to healthy controls but did not investigate nerve structures themselves [9, 20]. Therefore, we conducted this study with the aim to explore different MRI modalities that might be useful in future studies to assess and quantify muscle denervation and nerve degeneration in SMA.

## Methods

### Study design

Sixteen patients (six females, ten males; mean age 39.63 ± 2.82 years) with SMA and 13 age-matched healthy controls (four females, nine males; mean age 49.42 ± 3.7 years) were included in this study. All patients were diagnosed with SMA2 and 3 according to the guidelines of the Neuromuscular Disorder Society [1]. Four patients had 2–3 *SMN2* copies and seven patients 4–5 *SMN2* copies. The number of SMN2 copies was not applicable in five patients. Exclusion criteria were other neuromuscular diseases and contraindications against MRI. Clinical characteristics are shown in Table 2. All patients were on Nusinersen treatment (every 4 months, mean duration 10.9 ± 7.8 months). The local ethics committee approved the study, and all subjects gave written informed consent before study inclusion. This prospective, longitudinal, non-randomized, clinical, single-center study was carried out under the Declaration of Helsinki.

### Clinical assessment

Established clinical scores (HFMS, HFMSE, RULM and 6MWT) were performed for each patient at baseline (t0) and after 6 months (t1). Briefly, the HFMS is made up of 20 items in which the patients’ physical function is tested [21]. The expanded HFSM (HFMSE) was created for SMA patients who can sit and walk and is supplemented by 13 more items. The scores are used for measuring the severity and progression in SMA patients. In the scales, the items are subdivided into three functional categories (standing and transfers; axial and proximal motor function; distal motor function). The HFMS ranges from 0 (severe impairment) to 2 (no impairment) with a minimum total score of 0 and a maximum total score of 40. The HFMSE score ranges from 0 (severe impairment) to 2 (no impairment) per item with a minimum total score of 0 and a maximum total score of 66. The Revised Upper Limb Module (RULM) was established to assess motor performance of the upper limbs. The six-minute walk test (6MWT) evaluates functional exercise capacity and reflects motor fatigue in ambulatory SMA patients.

### MRI data acquisition and analysis

The MRI protocol was based on a protocol already established in studies of patients with chronic inflammatory demyelinating polyneuropathy or amyotrophic lateral sclerosis [14, 19]. The well-established protocol was extended by a quantitative T2 mapping sequence. As in the other studies, examinations were performed on a 3T whole-body MRI system (Ingenia, Philips Healthcare, Best, The Netherlands). With feet first in a supine position, participants were positioned so that their right thigh was examined deep within a knee coil (dStream T/R Knee 16ch Coil, Philips Healthcare, Best, Netherlands) with the center of the coil approximately 5–10 cm above the upper pole of the patella.

### MRI sequences

A SHINKEI-based three-dimensional T2-weighted turbo spin echo (3D T2 TSE) sequence with fat and vascular signal suppression was used to delineate the nerve [22–24]. Based on this planning sequence, an axial T2-weighted mDixon TSE (2D T2 TSE) for anatomic assessment and the DTI sequence were performed perpendicular to the sciatic nerve.

To quantify the intramuscular fat fractions of the quadriceps femoris (QFM) and biceps femoris (BFM) muscles, a six-echo multi-echo gradient echo sequence (mDixon Quant, Philips Healthcare, Best, The Netherlands) generating PDFF maps was acquired transversely. For quantitative assessment of intramuscular T2 relaxation times, a T2 map sequence was performed similarly. For detailed MRI parameters, see Table 1.

**Table 1:** MRI parameters

	3D T2 TSE^a^	2D T2 TSE^b^	PDFF mapping^c^	DTI^d^	T2 mapping
Encoding Repetition time (ms)	3D 2000	2D 2500	3D 10	2D 6500	2D 5112 (Act. TR)
Echo time (ms)	273	60	6 echoes (TE1 = 1.45, ΔTE = 1.1)	62	16 echoes (TE1 = 17, ΔTE = 17)
Flip angle (°)	90	90	3	90	90
Matrix	216 × 143 × 143	640 × 468	108 × 107	128 × 130	192 × 180
Resolution (mm^3^)	1.25 × 1.25 × 1.4	0.3 × 0.4 × 4	1.8 × 1.8 × 4	1.5 × 1.5 × 4	1 × 1 × 4
Slices	143	30	20	20	18
Gap	N/A	0	N/A	0	0.4
*b* values (s/mm^2^)	N/A	N/A	N/A	0 and 800	N/A
Scan time	2:30	5:00	1:05	9:00	2:13
Sense	2.5	2	1	2	2

^a^3D T2 TSE—three-dimensional T2 turbo spin echo sequence

^b^TSE—turbo spin echo

^c^PDFF—proton-density fat fraction mapping generated by a six-echo multi-echo gradient echo sequence

^d^DTI—Diffusion Tensor Imaging

### Data analysis

A senior radiologist (T. L.) evaluated the MR images. A second senior radiologist (K. L.) validated the measurements in a subgroup. DTI raw data post-processing and complete MRI analysis were performed using IntelliSpace Portal (IntelliSpace Portal 9.0, Philips Healthcare, Amsterdam, The Netherlands).

In the DTI sequence, the sciatic nerve was examined using six freehand drawn ROIs in six adjacent slices of color-coded fractional anisotropy (FA) images in correlation with the anatomical information of the *b* = 0 and 2D T2 TSE images. The mean of these six FA values was then determined to obtain the final FA value of each subject. Fiber tracking of the nerve was performed to depict the examined part of the thigh.

To determine the average intramuscular fat fractions and *T*_2_ times, respectively, subtotal ROIs were drawn freehand into each part of the quadriceps femoris muscle (vastus lateralis, intermedius, medialis, rectus femoris) and the short and long heads of the biceps femoris muscle on the most proximal slice in each of the PDFF and *T*_2_ maps. The ROIs were drawn within 2 mm of the muscle boundaries. The differing area sizes (A_i) of the individual ROIs (ROI_i with individual fat fractions (FF_i)) were taken into account using the formula FF_mean_over_ROIs = sum (A_i × FF_i)/sum (A_i), where the sum is the summation over all ROIs.

### Statistical analysis

For all statistical analyses, dedicated software was used (Statistics Package for Social Sciences (SPSS), v26, IBM, Armonk, NY, United States; Graph Pad Prism, v7, GraphPad Software, San Diego, CA, United States). A group comparison analysis was performed using the Mann–Whitney *U* test. The Kruskal–Wallis test was applied to compare more than two groups. Nonparametric Spearman’s correlation tests were used to assess correlations. For inter-rater reliability, intra-class correlation coefficients (ICC) were deemed indicative. A *p* value < 0.05 was considered statistically significant. Statistical analyses of the graphs depict the mean ± standard error of the mean.

## Results

### Clinical characteristics

Clinical characteristics of the participants are shown in Table 2. All patients received Nusinersen three times a year after four initial loading doses. Overall, the patients were moderately affected with a mean HFMS of 29.97 ± 3.53. The median of number of SMN2 copies was 4 (minimum 2, maximum 5, 25 percentile 3, 75 percentile 4). Controls showed no anamnestic or clinical signs of small or large nerve fiber affection.

**Table 2:** Clinical data

	SMA	Controls	*p* value
Sex (female:male)	6:10	4:9	
Age (years)	39.6 (2.8)	49.4 (3.7)	0.08 (n.s.)
Height (cm)	166.8 (2.3)	181.2 (2.4)	0.0005 (***)
Weight (kg)	63.19 (4.3)	76.77 (4.7)	0.034 (*)
BMI (cm/kg^2^)	22.48 (1.1)	22.80 (0.9)	0.94 (n.s.)
Therapy (Nusinersen)	16/16		
Treatment duration (months)	10.9 (7.8)		
Ambulatory vs. non-ambulatory	5:11		

*SMA* spinal muscular atrophy, Standard error of the mean (SEM) in brackets, *n.s.* not significant, *p < 0.05

### PDFF mapping

**Fig. 1:**
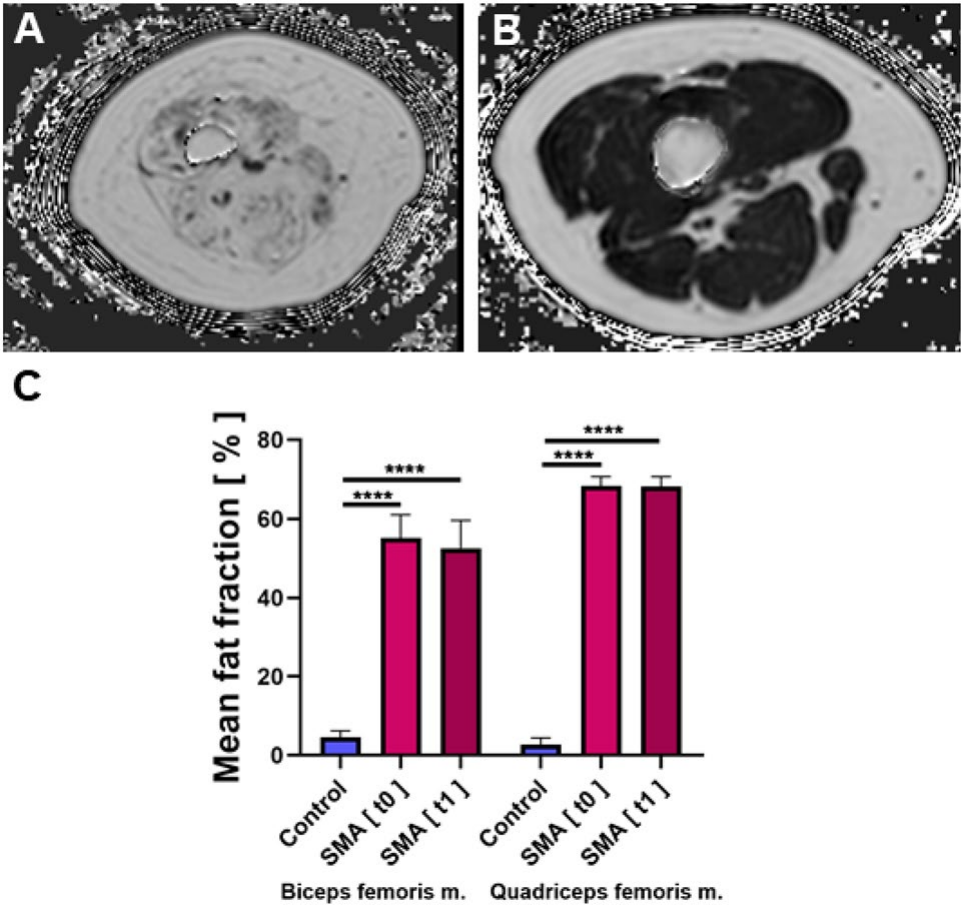
Dixon-based PDFF estimation. Representative Dixon-based PDFF maps of thigh muscles of a patient with SMA **A** and a healthy control **B**. Subtotal intramuscular ROIs were drawn on these maps to quantify the fat fraction. An increased fat fraction goes along with a higher intramuscular signal. **C** Depicts the average intramuscular fat fractions in the biceps femoris and quadriceps muscles of SMA patients and healthy controls. Fat fractions were significantly higher in both muscles in patients with SMA at baseline (*t*0) and after 6 months (*t*1). No significant differences between the fat fractions in SMA patients were detected at baseline and after 6 months

Spinal muscular atrophy (SMA) patients showed significantly higher intramuscular fat fractions in the QFM and in the BFM than healthy controls at baseline (*t* = 0) (68.41 ± 2.36 vs. 2.4 ± 1.73, *p* ≤ 0.0001 and 52.6 ± 7.03 vs. 4.23 ± 1.85, *p* ≤ 0.0001). Comparable values were measured after 6 months (*t* = 1) (68.21 ± 2.53 vs. 2.4 ± 1.73, *p* ≤ 0.0001 and 52.60 ± 7.03 vs. 4.23 ± 1.85, *p* ≤ 0.0001). No significant changes were seen in the intramuscular fat fractions in QFM and BFM in SMA patients after 6 months (68.41 ± 2.36 vs. 68.21 ± 2.53, *p* = 0.91; 55.14 ± 5.93 vs. 52.60 ± 7.03, *p* = 0.95, Fig. 1). Interrater reliability for PDFF mapping was excellent (ICC 0.981).

### Water *T*_2_ mapping

**Fig. 2:**
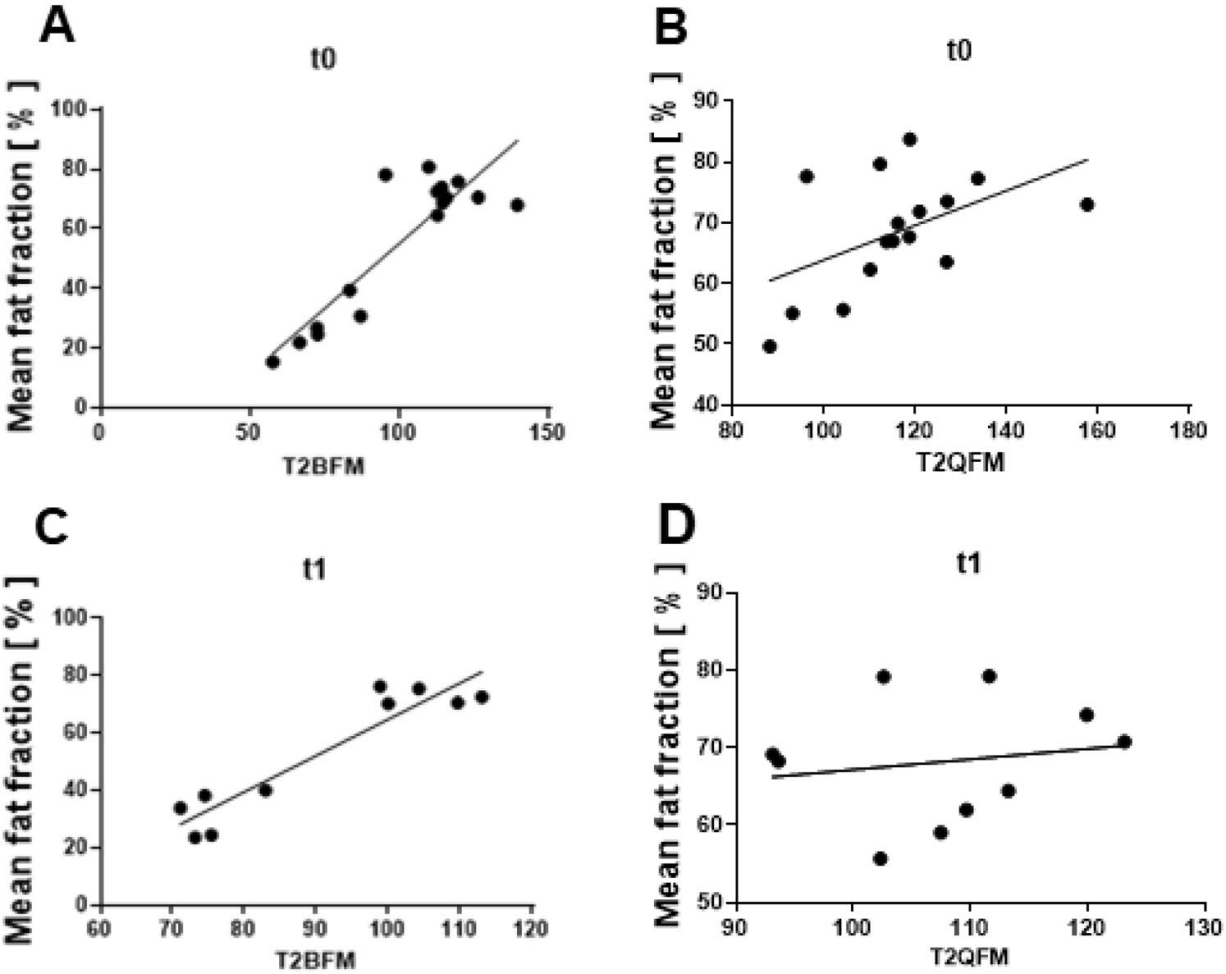
Correlation of Dixon-based PDFF mapping and *T*_2w_. Correlations between intramuscular fat fractions and *T*_2w_ in biceps (BFM) and quadriceps femoris muscles (QFM) in patients with SMA (**A**–**D**). Intramuscular fat fractions were positively correlated with T2 values in the two muscles, at baseline (*t*0) (**A**, **B**). After 6 months (*t*1) this positive correlation was sustained in the BFM (**C**), but not in the QFM (**D**)

T2BFM and the T2QFM correlated significantly positively at baseline with intramuscular fat fractions of BFM and QFM (*r* = 0.6294, *p* = 0.0106 and *r* = 0.5095, *p* = 0.0479, Fig. 2). At 6 months, the significant positive correlation between T2BFM and intramuscular fat fractions was replicable (*r* = 0.7818, *p* = 0.0105, Fig. 2). Interrater reliability for water *T*_2_ mapping was good (ICC 0.722).

### DTI

**Fig. 3:**
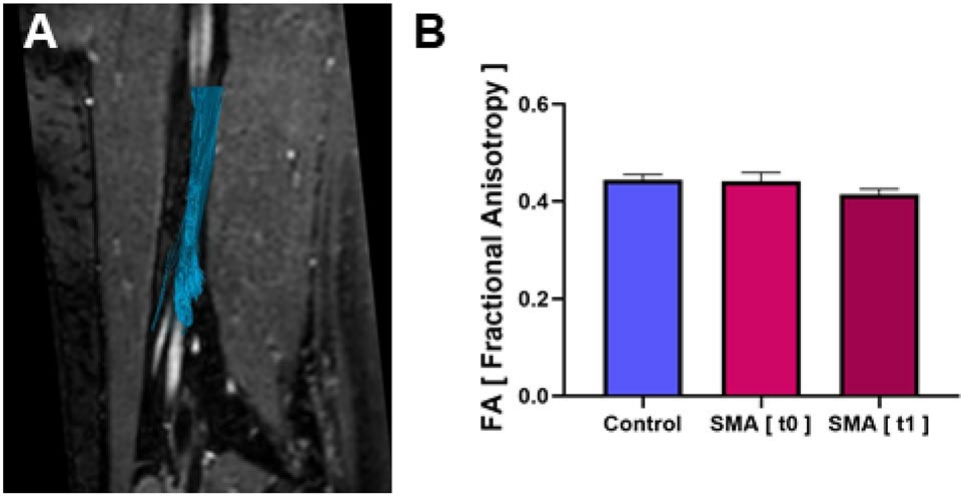
DTI. Tractography and fractional anisotropy (FA) in the proximal sciatic nerve segment of a healthy control. **A** illustrates FA sampling in a sagittal mid-section 3D T2 TSE-image of a healthy control. The course of the sciatic nerve is visualized by deterministic fiber tracking. **B **There were no significant differences seen in average FA values of sciatic nerves in patients with SMA and healthy controls at baseline (t0) and 6 months follow-up (t1)

DTI scans of the sciatic nerves of patients with SMA at baseline showed almost equal FA values compared to healthy controls (0.44 ± 0.1 vs. 0.44 ± 0.01, *p* = 0.77, Fig. 3). At 6 months, FA values were also not significantly different (0.41 ± 0.01 vs. 0.44 ± 0.01, *p* = 0.1186). No significant changes in FA in SMA patients were noted after six months (0.44 ± 0.1 vs. 0.41 ± 0.01, *p* = 0.35). Interrater reliability for DTI was good (ICC 0.715).

### Clinical correlations and scores

**Fig. 4:**
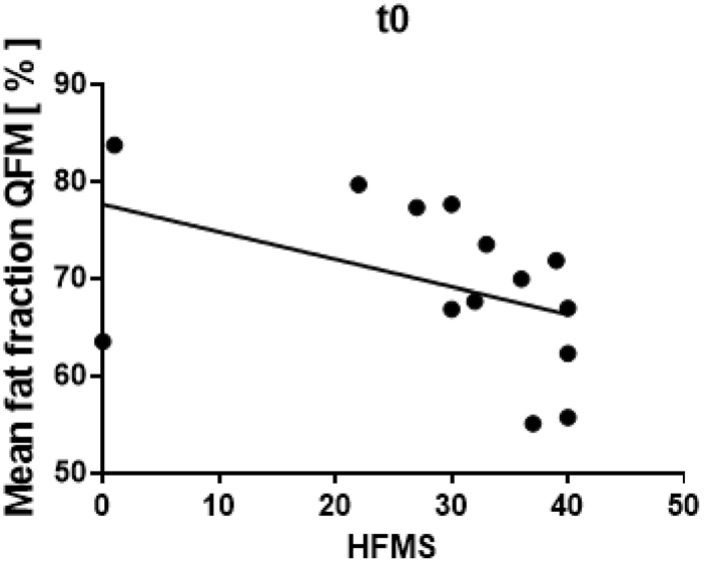
Correlation of Dixon-based PDFF mapping in QFM with HFMS. Intramuscular fat fractions in the quadriceps femoris muscle (QFM) of patients with SMA correlated significantly negatively with HFMS (*r* = − 0.5569, *p* = 0.0385)

MRI parameters were correlated with clinical measurements. The intramuscular fat fractions of the QFM at baseline significantly correlated negatively with the HFMS (*r* = − 0.5569, *p* = 0.0385, Fig. 4), i.e., a higher intramuscular fat fraction was associated with a lower HFMS. There was a non-significant trend for a negative correlation between HFMSE and the intramuscular fat fractions. No significant differences were seen between the MRI parameters and the number of SMN2 copies (data not shown).

### Subgroup analysis

**Fig. 5:**
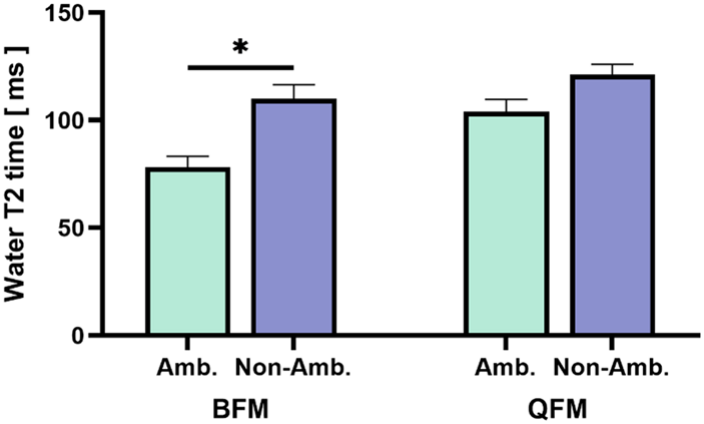
Non-ambulatory patients showed a higher T2 value in both muscles, in BFM and QFM, with a significant difference in the BFM (*p* = 0.0133). *Amb.* ambulatory SMA, *Non-Amb.* non-ambulatory SMA, *BFM* biceps femoris muscle, *QFM* quadriceps femoris muscle, *p < 0.05

In the PDFF mapping, non-ambulatory patients tended to have a higher mean fat fraction of the BFM and QFM than ambulatory patients (*n* = 11 vs. *n* = 5), but this effect was not statistically significant (data not shown). T2BFM in non-ambulatory patients was significantly higher compared to ambulatory patients (110.1 ± 6.46 vs. 78.18 ± 5.1, *p* = 0.0133, Fig. 5). A similar trend was observed in T2QFM but did not reach statistical significance. DTI FA values were not significantly different in ambulatory and non-ambulatory patients (0.4861 ± 0.02 vs. 0.4199 ± 0.02, *p* = 0.1533).

## Discussion

Out data suggest that adult SMA goes along with substantial intramuscular fat accumulation in thigh muscles, which Dixon-based PDFF and muscle water T_2_ mapping can quantify.

The study proves the feasibility of Dixon-based PDFF and water *T*_2_ mapping to detect pathological abnormalities in thigh muscles of SMA patients in line with previous studies using these measures as biomarkers of tissue fat concentration in different neuromuscular diseases [9, 12, 25].

We found significantly higher fat fractions in proximal muscles of SMA patients compared to controls. These results reflect the accentuated proximal muscle weakness in SMA. Our findings are in line with those of Otto and colleagues, who also reported increased intramuscular fat fractions in thigh muscles in SMA patients. However, compared to their cohort, we found even higher percentages of muscle fat (68.51% and 55.14% vs. 47.6%). These differences might reflect our study´s comparatively older patient cohort (mean age 39.6 vs. 30.2 years).

Intramuscular fat fractions of the QFM correlated significantly negatively with the HFMS at baseline. Other correlations between MRI biomarkers and clinical parameters could not be detected. This finding suggests that among all tested parameters, only the Dixon-based PDFF mapping may serve as a potential biomarker for disease severity in SMA patients, provided that larger studies spanning longer time periods may confirm correlations with functional decline over time. The lack of correlation with other parameters in our studies is explained by the overall high muscle fat infiltration of our patients and the small sample size. In addition, the different scales used here show different sensitivity for skills in ambulatory and non-ambulatory patients. For instance, the HFMS is an excellent tool for evaluation of motor function in non-ambulatory SMA patients, whereas the HFSME covers a broader spectrum of ambulatory functions.

The quantitative T2 values were also highly abnormal in SMA. This biomarker reflects pathophysiological changes of skeletal muscle tissue and is sensitive to different processes, such as inflammation, edema, myocytic lesions and necrosis [13]. Muscle denervation often simultaneously causes fatty and edematous alterations in one muscle [26], which both increase the T2 values. Therefore, the underlying changes cannot be further attributed to one or several pathological changes of muscle tissue. Our study demonstrates a positive correlation between T2 values and the intramuscular BFM and QFM fat fractions. Furthermore, non-ambulatory patients showed significantly higher T2 values in BFM compared to ambulatory patients. The most likely explanation for this finding is that the fatty degeneration confounds the T2 values. Since SMA is considered a systemic condition in which SMN protein depletion also affects the function of other tissues, including the skeletal muscle, heart, and autonomic nervous system [27], we cannot exclude that other pathological processes could affect MRI signaling properties of muscle in SMA patients. Water *T*_2_ mapping, as used here, is not an appropriate method to distinguish clearly between inflammatory processes and fatty infiltration. Elaborated methods with a reduced sensitivity to fatty infiltration for *T*_2w_ are needed [26].

Other studies announced a positive correlation between T2 intensity and mean annual increase of muscle fat replacement in late-onset Pompe disease [28] and GNE myopathy [29]. One has to consider, that higher intramuscular fat fractions might confound these data.

We did not find significant changes in PDFF mapping at baseline and during the course of 6 months. Potential explanations include that the period was too short or that the most patients were on treatment, preventing significant loss of spinal motor neurons. Carlier and colleagues demonstrated that in treatment-naive Pompe patients fatty infiltration progresses at a yearly rate of nearly 0.9% whereas this rate decreases to less than 0.68% during treatment. In our study, each patient received Nusinersen and showed a stable clinical course after six months. Another reason could be that Morbus Pompe is a myopathy with pathological processes primarily located in the muscles, whereas in SMA, mainly motoneurons are affected with secondary involvement of muscle tissue.

We did not find significant differences in FA values of the SMA patients compared to healthy controls at baseline and after six months. Previous studies have demonstrated that FA is a marker of axonal damage and regeneration [30]. The similar FA values in the two groups can probably be explained by the composition of the sciatic nerve, as it contains up to 70% sensory nerve fibers not involved in SMA pathogenesis [31]. It is conceivable that changes in FA levels in SMA would be more pronounced in motor nerves (e.g., the femoral nerve). There are studies that investigated DTI in other nerves such as the median nerve [32, 33]. However, the median nerve is also a mixed sensory and motor nerve and therefore not more promising when it comes to a motoneuron disorders such as SMA. To our knowledge, there are no experimental data of DTI in the femoral nerve in motoneuron conditions, most probably due to the fact, that the nerve itself is difficult to measure because of its small cross section. Another reason why we did not find significant differences in the FA values of SMA patients after 6 months may be the relatively short time period. As SMA is a slowly progressive disease it is expectable that changes in DTI could be detected after a longer time period, e.g., after a few years. Further studies are warranted to investigate this manner.

A limitation of our study is that we could not include treatment-naive patients, as all patients were already on nusinersen treatment for an average of 10.9 months. Consecutively, we could not collect reference data. Due to the fact, that newborn screening to SMA is explored and implemented in an increasing number of countries [34], it would be helpful to implement collaborations with pediatric centers to include therapy naive SMA patients to collect reference data.

In conclusion, this is the first longitudinal study to show the feasibility of a multi-parametric MRI protocol including Dixon-based PDFF mapping and water T2 mapping in thigh muscles as well as DTI in nerves in SMA. Our data demonstrate muscle atrophy going along with muscle fat replacement. Among the tested parameters, Dixon-based PDFF mapping and water T2 mapping appear to be more suitable to be further explored as SMA surrogate marker than DTI-based FA.
